# Insights into CSF-1/CSF-1R signaling: the role of macrophage in radiotherapy

**DOI:** 10.3389/fimmu.2025.1530890

**Published:** 2025-02-03

**Authors:** Qingchao Shang, Pei Zhang, Xiao Lei, Lehui Du, Baolin Qu

**Affiliations:** ^1^ Department of Radiation Oncology, The First Medical Center of Chinese PLA General Hospital, Beijing, China; ^2^ Department of Radiation Oncology, General Hospital of Southern Theatre Command of PLA, Guang Zhou, China

**Keywords:** CSF-1, macrophage, radiotherapy, radiation-induced pulmonary fibrosis (RIPF), tumor associate macrophages (TAM)

## Abstract

Macrophage plays an important role in homeostasis and immunity, and dysfunctional macrophage polarization is believed to be associated with the pathogenesis of tissue fibrosis and tumor progression. Colony stimulating factor-1 (CSF-1), a polypeptide chain cytokine, through its receptor (CSF-1R) regulates the differentiation of macrophages. Recently, the promising therapeutic potential of CSF-1/CSF-1R signaling pathway inhibition in cancer treatment is widely used. Furthermore, inhibition of CSF-1/CSF-1R signaling combined with radiotherapy has been extensively studied to reduce immunosuppression and promote abscopal effect. In addition, cumulative evidence demonstrated that M2 phenotype macrophage is dominant in tissue fibrosis and the inhibition of CSF-1/CSF-1R signaling pathway ameliorated pulmonary fibrosis, including radiation-induced lung fibrosis. Herein, we provide a comprehensive review of the CSF-1/CSF-1R signaling pathway in radiotherapy, with a focus on advances in macrophage-targeted strategies in the treatment of cancer and pulmonary fibrosis.

## 1 Introduction

Macrophages exist in all tissues of adult mammals and are considered to be components of resident tissues (1). Due to the important roles that macrophages play in both innate and adaptive immunity, they are involved in various pathological processes such as cancer and tissue repair (2, 3). Two major activated polarized states of macrophages are demonstrated in response to external environmental signals, and this polarized morphology is distinct from the irreversible tissue-specific phenotype of macrophages, which can be reversibly regulated by a variety of cytokines or transcription factors. Generally, macrophage polarization phenotypes can be classified into two types: M1 macrophages and M2 macrophages (4). M1 macrophages secret proinflammatory factors and show proinflammatory functions. M2 macrophages exhibit anti-inflammatory functions and are involved in tissue repair (5). The imbalance of inflammatory and anti-inflammatory macrophage phenotypes is critical in kinds of pathological processes, including the development of tissue fibrosis and tumor (6–8). Thus, modulation of macrophage polarization is a potential therapeutic strategy for a variety of diseases. Macrophage polarization is regulated by several factors, including CSF-1/CSF-1R signaling, which is proved to be critical for shaping the M1/M2 macrophage phenotype (9). CSF-1 promotes the differentiation of myeloid cells into macrophages through its receptor (CSF-1R), and regulates the migration, proliferation, survival and polarization of macrophages.

Radiotherapy has become one of the most important treatments for malignant tumors. According to statistics, about 60% of patients will receive radiotherapy (10). Studies have shown that radiotherapy combined with CSF-1R inhibitors exert synergistic effects through regulating macrophage polarization in the tumor microenvironment (TME), thereby improving tumor local control and enhancing anti-tumor immunity (11, 12). In addition, pulmonary fibrosis is a long-term complication of radiation-induced lung injury, in which M2-dominant phenotype is involved (13). Therefore, targeting polarization of macrophages to explore new strategies for the prevention and treatment of radiation-induced pulmonary fibrosis attract more and more attention in recent years.

Therefore, exploring the physiological and pathological roles of CSF-1/CSF-1R signal transduction in tumor treatment and radiation-induced pulmonary fibrosis is of great significance for the comprehensive treatment of malignant tumors. Here we summarize the biological functions of the CSF-1/CSF-1R signaling pathway and its role in radiotherapy.

## 2 CSF-1/CSF-1R signaling pathway: structure and biological function

CSF-1, also known as macrophage colony-stimulating factor (M-CSF), is a homologous dimer protein with three different forms: secreted glycoprotein, secreted glycoprotein polysaccharide, and a membrane-bound glycoprotein (14). CSF-1 is expressed in a variety of cells, such as osteoblasts and various cancer cells, which can be used as a potential tumor marker (15, 16). CSF-1R is a tyrosine kinase transmembrane receptor that is activated in an autocrine or paracrine manner. It is encoded by the oncogene *c-fms*, and its kinase domain contains 20 tyrosine residues, with a highly conserved structure (17). CSF-1R is highly expressed in a variety of tumor cells, including lung cancer, breast cancer, lymphoma, cervical cancer and so on (18). CSF-1 binds to its unique receptor CSF-1R in a hydrophilic manner, which triggers dimerization and phosphorylation of CSF-1R, leading to subsequent biological effects (19). Under physiological conditions, CSF-1 is capable of promoting macrophages polarized into M2 phenotype, which is involved in the latter phases for maintaining tissue repair and homeostasis (20, 21). In cancer, the chronic M2 phenotype or the tumor-associated macrophages (TAMs) inhibits tumor cell apoptosis, and induces angiogenesis under pathological conditions, thus promoting disease progression (22–25).

Accumulating evidence showed that, upregulated CSF-1 promotes the infiltration, survival, and metastasis of TAMs expressing CSF-1R in the tumor microenvironments (11, 26), and blocking the CSF-1/CSF-1R signaling pathway can reduce immunosuppressive TAMs in tumors. CSF-1/CSF-1R inhibitors have been identified as therapeutic targets for a variety of malignant tumors, such as glioma, hepatocellular carcinoma, breast cancer, lung cancer and pancreatic cancer (27), and have broad application prospects in tumor immunotherapy (**Table 1**).

**Table 1:** Curtent cilincal trails with inhibitors against CSF-1R in inflammatory diseases and cancer.

Target	Compound	Combination partners	Clinical Phase	Indications	NCT number	Reference (PMID)
CSF-1R	LY3022855(IMC-CS4)	Durvalumab, tremelimumab	I	Advanced solid cancers	NCT02718911	(60)
		/	I	Advanced solid cancers	NCT01346358	(61)
		/	I	Metastatic breast cancer, metastatic castration-resistant prostate cancer	NCT02265536	(62)
		cyclophosphamide, pembrolizumab, GVAX	I	Borderline resectable pancreatic cancer.	NCT03153410	/
		Vemurafenib (BRAF inhibitor), Cobimetinib(MEK inhibitor)	I,II	Advanced melanoma	NCT03101254	(63)
CSF-1R	Axatilimab(SNDX-6352)	Durvalumab(anti-PD-L1)	I	Solid Tumor	NCT03238027	/
		/	I,II	Chronic graft-versus-host disease	NCT03604692	(64)
		/	II	Chronic graft-versus-host disease	NCT04710576	(65)
		Durvalumab(anti-PD-L1)	II	Unresectable Intrahepatic Cholangiocarcinoma	NCT04301778	/
CSF-1R	Emactuzumab	Paclitaxel	I	Advanced solid tumors (diffuse-type giant cell tumor, soft tissue sarcoma or malignant mesothelioma, ovarian, endometrial,breast cancer, pancreatic cancer)	NCT01494688	(66)
		/	I	locally advanced diffuse-type tenosynovial giant cell tumours	NCT01494688	(67)
		Atezolizumab(anti-PD-L1)	Ib	Advanced solid tumors (urothelial bladder cancer and melanoma, non-small cell lung cancer)	NCT02323191	(68)
		Selicrelumab (agonistic cluster of differentiation 40 mAb)	Ib	Advanced solid tumor	NCT02760797	(69)
CSF-1R	JNJ-40346527(PRV-6527, Edicotinib)	/	I	Alzheimer Disease, Mild Cognitive Impairment	NCT04121208	/
		/	I/II	Relapsed or refractory classical Hodgkin lymphoma	/	(70)
		Disease-modifying antirheumatic drug	IIa	Active rheumatoid arthritis	NCT01597739	(71)
		/	IIa	Severely active Crohn’s disease	NCT03854305	/
CSF-1R	Cabiralizumab (FPA008, BMS986227)	APX005M (CD40 agonist), Nivolumab (anti-PD-1)	I	Biopsy-proven advanced melanoma, non-small cell lung cancer, or renal cell carcinoma	NCT03502330	(72)
		Nivolumab (anti-PD-1)	I	Advanced Solid Tumors	NCT02526017	(73)
		/	I/II	Pigmented Villonodular Synovitis, Tenosynovial Giant Cell Tumor	NCT02471716	/
		Nivolumab (anti-PD-1), HuMax-IL8	II	Head and Neck Squamous Cell Carcinoma	NCT04848116	/
		Nivolumab (anti-PD-1)	II	Peripheral T Cell Lymphoma	NCT03927105	/
CSF-1R	Vimseltinib(DCC-3014)	/	III	Tenosynovial giant cell tumour	NCT05059262	(74)
CSF-1R	ARRY-382(PF07265804)	Pembrolizumab (anti-PD-1)	II	Advanced Solid Tumors	NCT02880371	(75)
CSF-1R, KIT, FLT3	Pexidartinib(PLX3397)	/	I	Refractory leukemias or solid tumors including neurofibromatosis type 1-related plexiform neurofibromas	NCT02390752	(76)
		Durvalumab(anti-PD-L1)	I	Metastatic/​Advanced Pancreatic or Colorectal Cancers	NCT02777710	/
		Binimetinib	I	Advanced Gastrointestinal Stromal Tumor	NCT03158103	(77)
		/	I	Symptomatic, advanced solid tumors	NCT02734433	(78)
		/	I/II	Tenosynovial Giant-Cell Tumor	NCT01004861	(79)
		Sirolimus (mTOR)	I/II	Sarcoma, Malignant Peripheral Nerve Sheath Tumors	NCT02584647	(80)
		Pembrolizumab (anti-PD-1)	I/II	Melanoma, Non-small Cell Lung Cancer, Squamous Cell Carcinoma of the Head and Neck, Gastrointestinal Stromal	NCT02452424	/
		/	II	Recurrent glioblastoma	NCT01349036	(81)
		/	III	Pigmented Villonodular Synovitis, Tenosynovial Giant Cell Tumor	NCT02371369	(82)
CSF-1R, VEGFRs, FGFR-1	surufatinib	/	I	Healthy Chinese subjects	NCT02320409	(83)
		Toripalimab(anti-PD-1)	I	Advanced gastric/gastroesophageal junction adenocarcinoma, esophageal squamous cell carcinoma, biliary tract cancer	NCT04169672	(84)
		Toripalimab (anti-PD-1)	I	Advanced solid tumors	NCT03879057	(85)
		Toripalimab (anti-PD-1)	II	Advanced neuroendocrine tumours and neuroendocrine carcinomas	NCT04169672	(86)
		/	Ib/II	Advanced Well-Differentiated Neuroendocrine Tumors	NCT02267967	(87)
		Toripalimab (anti-PD-1), etoposide,cisplatin	Ib/II	Advanced small-cell lung cancer	NCT04996771	(88)
		/	II	Advanced or Metastatic Differentiated Thyroid Cancer and Medullary Thyroid Cancer	NCT02614495	(89)
		/	III	Advanced well-differentiated pancreatic and extrapancreatic neuroendocrine tumors	NCT02589821, NCT02588170	(90)
CSF-1R, Aurora B, VEGF, PDGFRα, c-Kit	Chiauranib	/	I	Refractory advanced solid tumor and lymphoma	NCT02122809.	(91)
CSF-1R, VEGFRs	SYHA1813	/	I	Recurrent high-grade gliomas or advanced solid tumors	ChiCTR2100045380	(92)
CSF-1R, PDGFR, VEGFR	Vorolanib(X-82)	Everolimus	I	Solid tumors	NCT01784861	(93)
CSF-1R, Bcr-Abl, DDR1	Nilotinib	/	II	Locally advanced pigmented villonodular synovitis	NCT01261429	(94)
CSF-1R, VEGFR2, PDGFRβ, c-kit, FLT3, RET	Sunitinib	/	II	Advanced germ cell tumor	NCT00912912	(95)
CSF-1R, cKIT, Lyn, Fyn, PDGFR	Masitinib (AB1010)	Isoquercetin	II	SARS-CoV 2, COVID-19, Coronavirus Disease 2019	NCT04622865	/
CSF-1R, VEGFR, cKIT, BRAF, PDGFR, FGFR	Regorafenib (Stivarga)	/	II	Hepatocellular Carcinoma	NCT04476329	/
		/	II	Malignant Solid Tumor	NCT04116541	/
CSF-1R, Aurora B	Chiauranib	Etoposide, paclitaxel	Ib,II	Recurrent ovarian cancer	NCT03901118 NCT03166891	(96)

In addition, aberrant expression of CSF-1/CSF-1R signaling has been reported in kinds of inflammatory diseases (12, 28–31). Related studies have shown that chronic inflammation largely leads to tissue fibrosis, including pulmonary fibrosis, in which M2 macrophages play an important role (32, 33). Previous data indicated that CSF-1 contributes to pulmonary fibrosis in mice. CSF1^-/-^ mice demonstrated less fibrosis in response to bleomycin challenge (34). And M2 macrophages are responsible for pulmonary fibrotic disease in many fibrosis models. Although the pathogenesis of pulmonary fibrosis remains unclear, abnormalities in lung macrophages have been reported to dramatically contribute to the pathogenesis of pulmonary fibrosis. Blocking the CSF-1/CSF-1R signaling pathway may be a potential therapeutic target for pulmonary fibrosis (35, 36).

## 3 CSF-1/CSF-1R signaling pathway in tumor radiotherapy

TME includes cells such as macrophages, dendritic cells, T cells, endothelial cells and fibroblasts, as well as extracellular matrix (ECM) components, proteases, and cytokines, playing a critical role in tumor evolution and metastasis (37). In the tumor growth and metastasis, immune cells in the tumor microenvironment play very important roles, including T lymphocytes, B lymphocytes, TAMs, bone marrow-derived suppressive cells and other cells. TAMs are the immune cells that infiltrate the most tumor tissue. They are immune regulatory cells that differentiate from peripheral monocytes under the influence of the tumor microenvironment. TAMs are mainly M2-polarized macrophages, which play immunosuppressive roles, promoting tumor growth, metastasis, and angiogenesis (38). TAMs are closely related to CSF-1. When tumor cells proliferate uncontrollably, they secrete a large amount of CSF-1, stimulating the production of a large number of immunosuppressive M2 macrophages (39).

TAMs are associated with radiation resistance, and CSF-1/CSF-1R inhibitors can alter macrophage polarity and show radiation sensitization effects. Radiotherapy can induce adaptive immune responses in tumors, which can be achieved by increasing the expression of MHC class I proteins in tumor cells, enhancing antigen presentation, promoting the release of damaging molecular-related patterns from damaged tumor cells and enhancing the recruitment and activity of antigen-presenting cells (40), and the combination of CSF-1/CSF-1R inhibitors and radiotherapy exert a synergistic effect and enhance the anti-tumor immunity.

Jones et al. demonstrated that radiotherapy combined with macrophage depleting agents enhanced tumor killing and found that colorectal (MC38) and pancreatic (KPC) cell lines produced CSF-1 after irradiation, leading to an increase in M2 macrophages, which were immunosuppressive in tumors. The use of CSF-1 monoclonal antibodies reversed macrophage aggregation and inhibited tumor growth (41). Parsons and colleagues found that the differentiation of hematopoietic stem cells and progenitor cells into M2 macrophages within tumors promoted tumor growth after radiotherapy. Using a non-small cell lung cancer-bearing mouse model, the tumor was irradiated alone or in combination with the CSF-1R inhibitor GW2580 (selective blockade of CSF-1R self-phosphorylation and activation) for 20 Gy. They found that the CSF-1/CSF-1R signaling pathway induced hematopoietic progenitor cells and stem cells derived from bone marrow within the tumor differentiating into M2 tumor-associated macrophages, which contributed to tumor survival and regeneration after radiotherapy. The use of GW2580 improved the tumor-killing ability of radiotherapy and the survival rate of mice (42). Seifert et al. studied the inhibitory immune response of macrophages induced by radiotherapy in a mouse model of pancreatic cancer. Radiation induced an increase in the infiltration of M2 macrophages in pancreatic tumor tissue and reduced the anti-tumor effect mediated by T cells. Combination of radiotherapy with CSF-1 monoclonal antibody changed the phenotype of macrophages in pancreatic tumors, enhanced the effect of T cells, and slowed down the growth of tumor (43). Stafford et al. investigated the effect of inhibiting CSF-1R on bone marrow cell recruitment and polarization to delay the recurrence of glioblastoma after radiotherapy. They constructed a mouse model of intracranial *in situ* glioblastoma and administered whole brain irradiation at a dose of 12 Gy or in combination with PLX3397 (a small molecule CSF-1R tyrosinase activity inhibitor). They found that combined treatment enhanced the response of intracranial tumors to radiotherapy and blocked the differentiation of mononuclear cells recruited by radiotherapy into immunosuppressive and angiogenic TAMs, thereby delaying tumor recurrence (44). Xu et al. found that blocking CSF-1R signaling pathway inhibited tumor infiltration of bone marrow cells and improved the radiotherapy efficacy of prostate adenocarcinoma. The authors constructed a tumor-bearing animal model of prostate cancer with local irradiation of 15 Gy and found that the expression level of CSF-1 and the recruitment of tumor-infiltrating bone marrow cells were increased. Further mechanism investigation showed that radiotherapy induced the recruitment of kinase ABL1 to the nucleus by DNA damage where it bound to the CSF1 gene promoter and enhanced the transcription of the CSF1 gene. Elevated CSF-1 played a crucial role in the systemic recruitment of primary myeloid cells to the irradiated tumor. Combined with CSF1R inhibitors, it reduced the number of tumor-infiltrating bone marrow cells and inhibited tumor growth (45). In the tumor-bearing model of breast cancer, combination of CSF-1 monoclonal antibody or PLX3397 would lead to depletion of immunosuppressed macrophages, significantly delaying tumor regeneration after radiotherapy (46). In the future, CSF-1/CSF-1R pathway inhibitors combined with radiotherapy are potential targets for the treatment of tumors.

## 4 CSF-1/CSF-1R signaling pathway in radiation-induced pulmonary fibrosis

60-70% of cancer patients require radiotherapy during treatment, and the most common and severe side effect after thoracic radiation is radiation-induced lung injury. Up to 50% of lung cancer patients develop pneumonia in the high-dose areas of the lungs, and pulmonary fibrosis occurs in 70-80% of patients (47). Radiation-induced pulmonary fibrosis is a progressive, interstitial fibrosis pulmonary disease, which is an important pathological process in the late stage of radiotherapy. Its clinical manifestations are mainly characterized by progressive dyspnea and decreased lung function (48). There are no effective medications for the treatment of radiation-induced pulmonary fibrosis by now (49).

Although the pathogenesis of pulmonary fibrosis is still unclear, recent studies have reported the important role of macrophages as key regulatory factors for fibrosis (50), with M2 polarized macrophages playing an important role in various fibrosis models (51–53). Depletion of macrophages, especially M2-type macrophages, attenuated pulmonary fibrosis (54, 55). In addition, studies showed that CSF-1 was elevated in alveolar lavage in patients with pulmonary fibrosis and stimulated macrophages and fibroblasts to participate in fibrosis formation (56). As an essential factor for macrophage differentiation and proliferation, the inhibition of CSF-1/CSF-1R pathway affects macrophage production and thus attenuates lung fibrosis.

Baran et al. investigated the role of CSF-1 in the pathogenesis of pulmonary fibrosis and found that its expression was elevated in patients with pulmonary fibrosis. In mouse model of bleomycin-induced pulmonary fibrosis, knocking out CSF-1 or usage of CSF-1R inhibitor showed protective effect (56). Zhou et al. investigated the role of IL-34 (a ligand of CSF-1R) and found that IL-34 upregulated IL-6 and IL-8 expression in human lung fibroblasts and these effects were reversed when treated cells with anti-CSF-1R antibody. These data confirmed the inflammatory effect of IL-34 on human lung fibroblasts and suggested that the IL-34/CSF-1R axis may be a novel therapeutic target in pulmonary disease (57). Joshi et al. investigated that monocyte-derived alveolar macrophages in the pulmonary fibrosis microenvironment were regulated by the CSF-1/CSF-1R signaling pathway. In a mouse model of asbestos-induced pulmonary fibrosis, CSF-1 monoclonal antibodies or inhibitor PLX3397 were used to block the CSF-1/CSF-1R signaling pathway to reduce monocyte differentiated alveolar macrophages and the alleviated pulmonary fibrosis was observed (58).

The evidence of CSF-1/CSF-1R signaling pathway in radiation-induced pulmonary fibrosis is limited. Zhang et al. showed that in the middle and late stages of radiation-induced lung injury (RILI), various pro-fibrotic cytokines such as IL-4 and IL-13 promoted macrophage polarization into M2 macrophages, leading to excessive secretion and deposition of extracellular matrix, ultimately resulting in fibrosis and structural changes (52). M2 macrophages can regulate myofibroblast activity in the middle and late stages of RILI through TGF-β/Smad pathway, promoting the progression of radiation-induced pulmonary fibrosis (59). Meziani et al. studied the use of CSF-1R inhibitors to prevent radiation-induced pulmonary fibrosis by depleting pulmonary interstitial macrophages and constructed a 16Gy mouse chest irradiation model. They found that using clodrosomes to deplete alveolar macrophages did not improve pulmonary fibrosis while using CSF-1R monoclonal antibodies to deplete pulmonary interstitial macrophages alleviated radiation-induced pulmonary fibrosis (59). Despite the rapid advances in radiation oncology in plan design and image-guided radiotherapy, normal tissue toxicity remains a dose-limiting factor for optimal local tumor control. The inhibition of the CSF-1/CSF-1R signaling pathway offers a novel therapeutic modality for mitigating radiation-induced pulmonary fibrosis.

## 5 Summary

**Figure 1:**
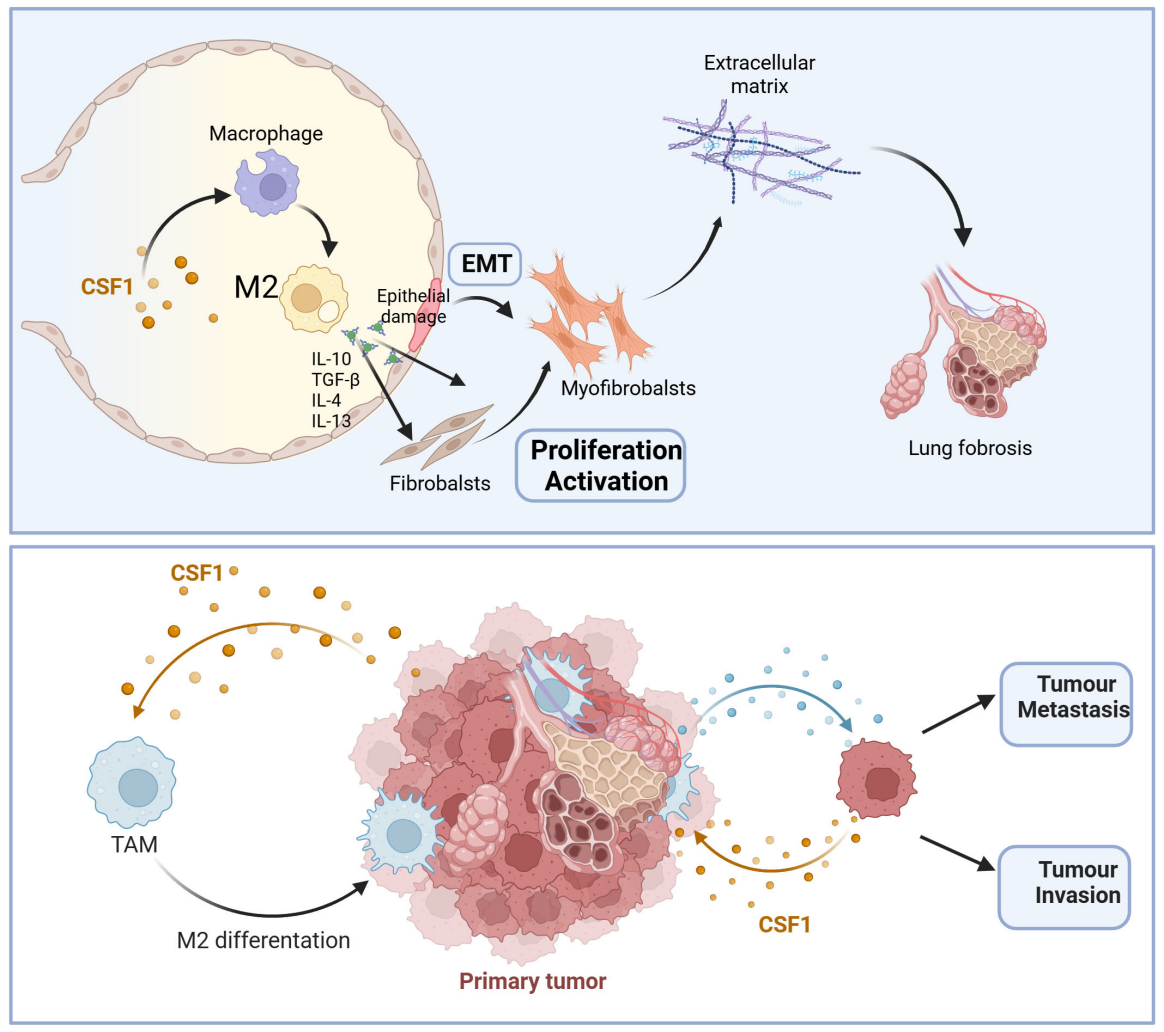
Schematic overview of critical role of CSF-1/CSF-1R signaling pathway in pulmonary fibrosis and tumor progression. IL-10, Interleukin-10; IL-4, Interleukin-4; TGF-β, Transforming growth factor-β; IL-13, Interleukin-13; EMT, Epithelial-mesenchymal transition; M2, alternatively activated macrophages.

In summary, due to the crucial regulatory role of the CSF-1/CSF-1R signaling pathway in tumor development and fibrotic processes, inhibiting the CSF-1/CSF-1R signaling pathway seems to be a promising strategy for cancer treatment and fibrosis (**Figure 1**). Although different experimental models may yield some controversial results, the accumulation of preclinical evidence has paved the way for the clinical application of CSF-1/CSF-1R inhibitors in tumor radiotherapy and fibrotic diseases. Further researches are needed to achieve further understanding of the interaction between CSF-1/CSF-1R signaling pathway inhibitors and tumor radiotherapy and fibrosis. This will help provide new treatment strategies for precise radiotherapy of tumors and reduce radiotherapy side effects.
